# Early-life sugar rationing, brain aging, and long-term neurodegenerative and psychiatric health outcomes: a population-based natural experiment study

**DOI:** 10.1038/s41514-026-00452-z

**Published:** 2026-07-22

**Authors:** Xingji Lian, Amei Chen, Xiaoyan Zhu, Yuyu Huang, Chongzhe Yang, Yan Huang, Meilong Ran, Longyan Yin, Bing Zhang

**Affiliations:** 1 Department of Geriatrics, National Key Clinic Specialty, Guangzhou First People’s Hospital, Guangzhou Medical University, Guangzhou, China; 2 Department of Radiology, Guangzhou First People’s Hospital, Guangzhou Medical University, Guangzhou, China; 3 Suzhou Center for Disease Prevention and Control, Suzhou, China

**Keywords:** Diseases, Medical research, Neurology, Neuroscience, Risk factors

## Abstract

Early-life nutrition is linked to adult brain health, yet evidence from quasi-experimental studies remains scarce. Leveraging the UK’s historical sugar rationing as a natural experiment, we examined whether early-life sugar restriction reduces the risk of neurodegenerative and psychiatric disorders in later life. Among 60,394 UK Biobank participants (born 1951–1956), three groups were defined: in-utero-only, in utero plus 2 years, and non-rationed controls. Compared with non-rationed individuals, those exposed to sugar restriction during the first 1000 days after conception, showed significantly lower risks of all-cause dementia (by 27%), Alzheimer’s disease (46%), depression (11%), and anxiety (20%). Dose-response analyses revealed stronger protective associations against depression and anxiety when postnatal sugar restriction extended beyond 6 months of age. Sex-stratified analyses showed consistent protective effects in women for Alzheimer’s disease, depression, and anxiety. Neuroimaging revealed decelerated brain aging, reflected by a 0.39-year reduction in the brain age gap, alongside structural changes in reduced gray–white matter contrast and larger subcortical volumes, including the hippocampus and thalamus. Minimal associations were observed for in‑utero‑only exposure. These quasi-experimental findings suggest that early-life sugar restriction is associated with attenuated neurobiological aging and lower risks of dementia and psychiatric disorders, highlighting sugar intake as a potential modifiable factor of lifelong brain health.

## Introduction

The escalating global burden of neurological and psychiatric disorders, such as dementia, depression, and anxiety, imposes substantial societal and economic costs due to lifelong disability and increased healthcare expenditures^1^. The Developmental Origins of Health and Disease (DOHaD) framework proposes that early-life exposures, particularly during the first 1000 days (conception to 2 years of age) of brain development, are critical determinants of long-term health^2–5^. Nutrition is a key factor within this window, yet modern dietary patterns raise concerns: pregnant and breastfeeding women currently consume more than triple the recommended amount of added sugars, and many commercial baby foods derive a substantial proportion of their calories from added sugars^6–8^. These dietary patterns position early-life sugar intake as a potentially modifiable risk factor for later brain health.

Accumulating evidence suggests that high sugar exposure in early life may harm the developing brain. Biological studies indicate that high sugar exposure may promote inflammation and disrupt stress-response systems, potentially leading to cognitive and behavioral problems^9–11^. Observational studies have linked poor maternal metabolic health, for example, elevated blood glucose, to increased risks of attention-deficit/hyperactivity disorder and anxiety in children^4,12^, and these findings are supported by animal research^13,14^. Despite this biological plausibility, direct causal evidence that early-life sugar restriction improves long-term brain health remains lacking, largely owing to ethical and practical constraints that preclude randomized trials during this vulnerable phase.

To overcome this fundamental methodological challenge, we leveraged a historical natural experiment: the UK sugar rationing policy (1942–1953). This policy significantly restricted sugar availability, and notably, no sugar ration was allocated to children under two years of age^15–17^. Its abrupt end in September 1953 triggered an immediate near-doubling of average adult sugar consumption, while intake of other major foods remained stable. This provided a clear quasi-experimental contrast in early-life sugar exposure between cohorts born just before and after the policy ended, substantially reducing confounding from broader socioeconomic trends and enabling stronger causal inference. Leveraging this natural experiment within the UK Biobank, we assessed the risks of multiple neurodegenerative and psychiatric disorders, including dementia, Alzheimer’s disease (AD), anxiety, and depression, by comparing individuals exposed to sugar rationing during the first 1000 days post-conception with those exposed to higher sugar levels after rationing ended. Additionally, we incorporated markers of brain age gap (BrainAGE) and subclinical structural alterations, captured by magnetic resonance imaging (MRI) indices, to examine the link between early life sugar rationing and long-term brain health. We hypothesized that early-life sugar rationing would reduce the risk of these disorders and decelerate brain aging, with longer exposure conferring greater neuroprotection in later life.

## Results

### Participant characteristics

Table 1 summarizes the characteristics of the 60,394 participants born between October 1951 and March 1956, of whom 43.8% were male. They were categorized by early‑life sugar rationing exposure into three groups: in‑utero-only (*N* = 9963, born October 1953 to June 1954, representing those exposed solely gestation), first-1000-day exposure 1000 days (*N* = 38,227, born October 1951 to June 1954, encompassing those with any exposure during the first 1000 days, including the in-utero-only subgroup), and non‑rationed controls (*N* = 22,167, born July 1954 to March 1956, with no exposure). With the exception of a higher proportion of White ethnicity and parental history of dementia in the first‑1000‑day exposure group, baseline covariates (sex, survey year, parental history of Parkinson’s disease [PD], parental history of depression, and all polygenic risk scores [PRS]) were well balanced across groups (all *P* > 0.05).

**Table 1:** Baseline characteristics of participants born between October 1951 and March 1956 in the UK Biobank

	Not rationing	Rationing in utero	Rationing up to 1000 days	*P* values
*N*	22,167	9963	38,227	
Sex, Male (%)	9667 (43.6)	4382 (44.0)	16,776 (43.9)	0.752
Ethnic, White (%)	21,976 (99.1)	9897 (99.3)	37,998 (99.4)	0.001
Birthplace (%)				0.001
England	18,878 (85.2)	8570 (86.0)	32,847 (85.9)	
Wales	1085 (4.9)	452 (4.5)	1930 (5.0)	
Scotland	2204 (9.9)	941 (9.4)	3450 (9.0)	
Survey year (%)				0.272
2007	2307 (10.4)	1000 (10.0)	3857 (10.1)	
2008	8612 (38.9)	3878 (38.9)	14,904 (39.0)	
2009	7620 (34.4)	3540 (35.5)	13,279 (34.7)	
2010	3628 (16.4)	1545 (15.5)	6187 (16.2)	
Educational attainment, *n* (%)				<0.001
College education	8049 (36.3)	3560 (35.7)	13,674 (35.8)	
Vocational qualification	921 (4.2)	443 (4.4)	1855 (4.9)	
Any school degree	10,678 (48.2)	4737 (47.5)	17,848 (46.7)	
Other	2519 (11.4)	1223 (12.3)	4850 (12.7)	
TDI, mean (SD)	−1.44 (2.99)	−1.53 (2.97)	−1.52 (2.95)	0.003
Parents with PD (%)	2408 (10.9)	1148 (11.5)	4394 (11.5)	0.046
Parents with depression (%)	2295 (10.4)	1018 (10.2)	3875 (10.1)	0.699
Parents with dementia (%)	2574 (11.6)	1237 (12.4)	5095 (13.3)	<0.001
PRS for AD, mean (SD)	0.06 (1.00)	0.05 (1.00)	0.06 (1.00)	0.515
PRS for PD, mean (SD)	−0.15 (1.02)	−0.15 (1.02)	−0.15 (1.02)	0.996
PRS for schizophrenia, mean (SD)	−0.34 (1.01)	−0.33 (0.99)	−0.34 (0.99)	0.6
PRS for bipolar disorder, mean (SD)	−0.24 (1.04)	−0.24 (1.04)	−0.25 (1.04)	0.853
PRS for type 1 diabetes, mean (SD)	0.06 (1.09)	0.05 (1.09)	0.06 (1.10)	0.609
PRS for type 2 diabetes, mean (SD)	−0.14 (0.97)	−0.13 (0.96)	−0.14 (0.96)	0.794

*TDI* Townsend Deprivation Index, *SD* standard deviation, *PD* Parkinson’s disease, *AD* Alzheimer’s disease, *PRS* polygenic risk score.

### Longitudinal association between early-life sugar rationing and brain health

**Fig. 1:**
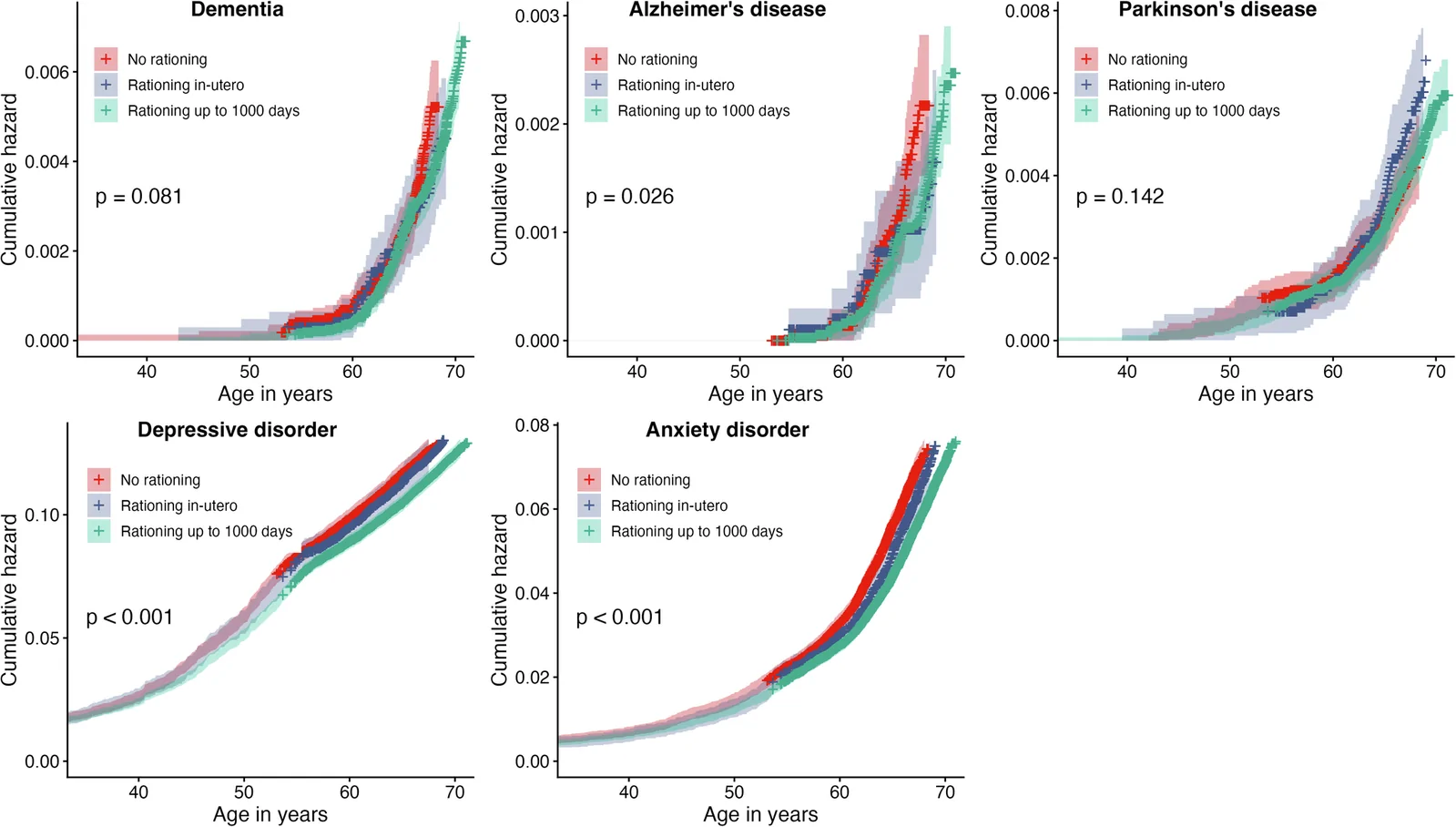
The cumulative risk of incident brain disorders stratified by exposure to rationing. Plots depict the crude cumulative risk and its 95% confidence interval of brain disorders, stratified by rationing pattern (no rationing, red; rationing in utero, blue; rationing up to 1000 days, green). *P* values were obtained using the log-rank test to examine differences between survival curves.

**Fig. 2:**
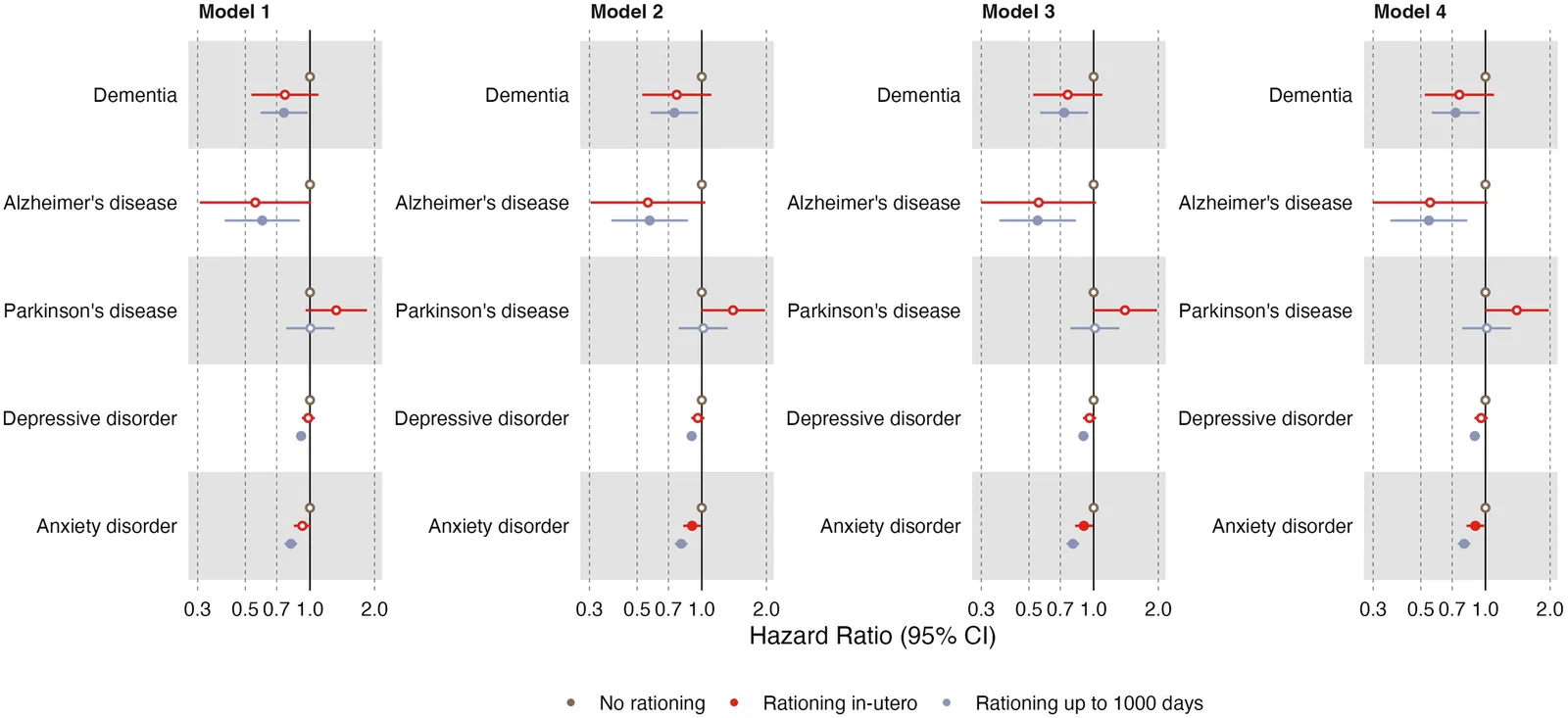
Association between rationing pattern and incident brain disorders in models with different sets of covariates. Participants from the UK Biobank born between October 1951 and March 1956, and aged 51–66 years at the time of survey, were included. Adults conceived within the 1000 days prior to September 1953 were classified as “rationing up to 1000 days” (born October 1951 to June 1954), and those conceived after this period as “non-rationed” (born July 1954 to March 1956). Adults exposed to rationing in-utero-only were those born between October 1953 and June 1954. Dots represent hazard ratio (HR) for adults exposed to rationing in utero or in utero plus 24 months after birth, relative to the reference group (non-rationed). The crude model (Model 1) did not adjust for any covariates. Model 1 was adjusted for calendar month of birth, survey year, deciles of north and east birth coordinates, place of birth (England, Wales, or Scotland), self-reported sex, and race (White vs. non-White). Model 2 was additionally adjusted for genetic risks, including parental history of brain diseases (depression, dementia, or Parkinson’s disease) and polygenic risk scores for brain disorders (dementia, Parkinson’s disease, schizophrenia, and bipolar disorder) and diabetes (type 1 and type 2 diabetes). Model 4 was further adjusted for the Townsend Deprivation Index and educational attainment. Dots represent HR, and horizontal lines indicate 95% confidence interval. Solid dots indicate statistically significant HR values below 1, while hollow dots represent non-significant HR values below 1.

During follow-up, a total of 307 participants were diagnosed with dementia, 123 with AD, 290 with PD, 7015 with depression disorder, and 4011 with anxiety disorder. Unadjusted Kaplan–Meier survival curves showed accelerated age‑related risk in those with no rationing exposure, except for PD and all-cause dementia (Fig. 1; event counts in Figs. S2–S6). Cox regression models revealed that extended sugar rationing during the first 1000 days conferred the strongest neuroprotection: versus non‑rationed controls, fully adjusted hazard ratios (HRs) was 0.73 (95% confidence intervals (CI): 0.56–0.94) for all-cause dementia, 0.54 (95%CI: 0.36–0.82) for AD, 0.89 (95%CI: 0.85–0.94) for depression, and 0.80 (95%CI: 0.75–0.85) for anxiety (Fig. 2). In‑utero‑only rationing was associated solely with reduced anxiety risk (HR = 0.90, 95%CI: 0.82–0.99). No significant associations were observed for PD. These estimates remained stable across nested models (Table S3). Dose-response analyses showed that longer postnatal rationing was associated with progressively lower psychiatric risk. Beyond 6 months of postnatal rationing, risk reductions became more pronounced; by 12 months, the HRs were 0.77 (95% CI: 0.71–0.84) for depression and 0.72 (95% CI: 0.65–0.80) for anxiety, with the strongest protection at 24 months (Fig. S7). No clear dose-response trends were observed for all-cause dementia, AD, or PD.

Sex-stratified analyses revealed differential associations. The most consistent protective associations were observed in females with exposure during the first 1000 days, who showed significantly reduced risks for AD (HR = 0.50, 95% CI: 0.29–0.87), depression (HR = 0.88, 95% CI: 0.83–0.94), and anxiety disorders (HR = 0.83, 95% CI: 0.76–0.90) (Table S4). In males, a significant risk reduction was observed only for anxiety disorders, with protective associations seen for both in utero exposure (HR = 0.77, 95% CI: 0.65–0.91) and with extended exposure during the first 1000 days (HR = 0.75, 95% CI: 0.67–0.83). No significant beneficial associations were found for all-cause dementia, AD, or depression in males, nor for PD in either sex (Table S4).

### Impact of early-life sugar rationing on neuroimaging phenotypes of BrainAGE

**Fig. 3:**
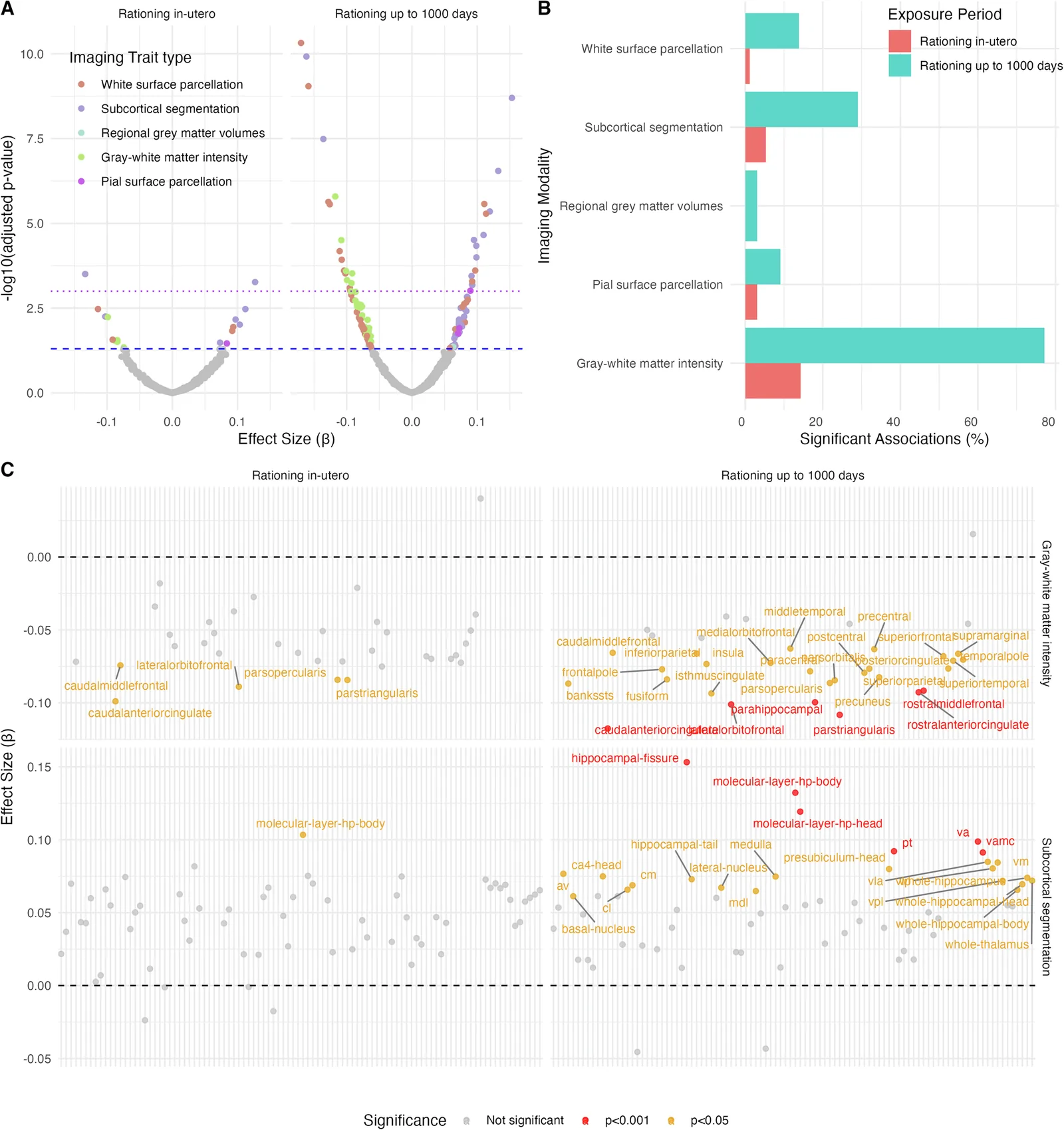
Associations between early-life sugar rationing exposure and multimodal brain imaging phenotypes. Volcano plot (**A**) showing the effect sizes (β) and statistical significance (−log10 of FDR-adjusted *p* values) for all tested associations between exposure to sugar rationing (in utero: red; up to 1000 days: blue) and brain imaging traits. Each point represents a distinct imaging phenotype, colored by the imaging category. Horizontal dashed lines indicate significance thresholds (*p*adj = 0.05 and 0.001). Bar plot (**B**) displays the proportion of significant associations (*p*adj < 0.05) within each imaging category, stratified by exposure period. **C** focuses on two imaging categories: gray–white matter intensity and subcortical segmentation, which yielded the highest number of significant findings. Each row corresponds to a specific imaging trait; points are colored by significance level, and traits with *p*adj < 0.05 are labeled with their original variable names. All associations were derived from linear regression models adjusted for age, sex, head motion, scanner position, calendar month of birth, deciles of north and east birth coordinates, place of birth, race, Townsend Deprivation Index, educational attainment, genetic risks for brain diseases, and total intracranial volume (for volumetric traits); *p* values were corrected for multiple testing using the Benjamini–Hochberg FDR procedure within each imaging category.

Significant associations between early-life sugar rationing and neuroimaging phenotypes were predominantly observed in the first-1000-day rationing group, with minimal findings observed for in-utero-only exposure (Fig. 3). Rationing during the first 1000 days was associated with widespread reductions in gray–white matter intensity contrast across approximately 70% of tested cortical regions, alongside larger subcortical volumes including the hippocampus, thalamus, and putamen. In contrast, the in-utero-only group showed few significant associations across all imaging modalities (Fig. 3). In subcortical segmentation analyses, rationing during the first 1000 days was associated with 32.1% of the volumetric measures assessed, including larger volumes of the putamen, pallidum, hippocampus, and thalamus (β range: 0.06–0.11, *P*_adj_ < 0.05). In comparison, in utero exposure was associated with only 4.6% of these measures. The most pronounced effects were observed for gray–white matter intensity contrast, where rationing during the first 1000 days was linked to widespread reductions across 71.4% of tested cortical regions (β range: −0.10 to −0.06, *P*_adj_ < 0.05), including the banks of the superior temporal sulcus, insula, and orbitofrontal cortex. No significant associations were found for the inutero-only group. For white-surface parcellation (Figs. S8–S10), rationing during the first 1000 days yielded 9.6% significant findings, such as increased insular area and paracentral region area, alongside reduced cortical thickness in multiple regions (β range: −0.15 to −0.11), whereas in utero exposure showed only 0.8% significant associations. Minimal associations were observed for pial-surface parcellation and regional gray matter volumes (3.0–6.1%), all within the first-1000-day rationing group. Of note, because the neuroimaging subsample and the brain age training sample both excluded individuals with pre‑existing neurological or psychiatric conditions, we did not adjust for depressive or anxiety disorders in these analyses.

**Fig. 4:**
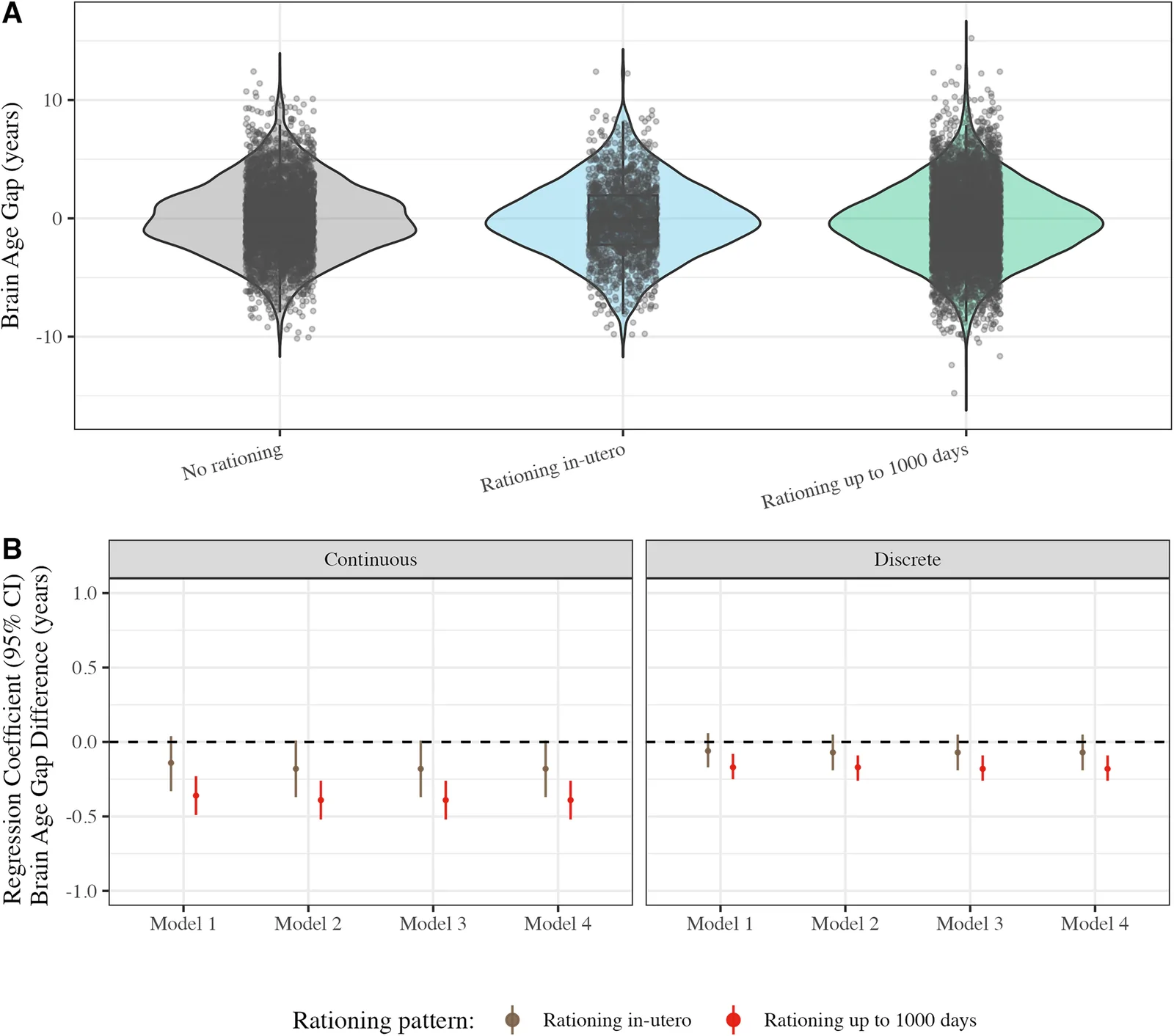
Brain age gap by early-life sugar rationing exposure and adjusted regression estimates. **A** Violin plots showing the distribution of brain age gap (BrainAGE) across three exposure groups. Compared with non-rationed controls, individuals exposed to rationing during the first 1000 days exhibited a left-shifted distribution, indicating younger-appearing brains. **B** Regression coefficients (95% confidence intervals) for the association between rationing exposure and BrainAGE. In both continuous (left) and discrete (right) brain aging outcomes, people exposed torationing up to 1000 days was associated with a significantly decelerated BrainAGE (red dots), whereas in-utero-only exposure showed weaker, non-significant trends (brown dots). The crude model (Model 1) did not adjust for any covariates. Model 2 was adjusted for calendar month of birth, survey year, deciles of north and east birth coordinates, place of birth (England, Wales, or Scotland), self-reported sex, and race (White vs. non-White). Model 3 was additionally adjusted for genetic risks, including parental history of brain diseases (depression, dementia, or Parkinson’s disease) and polygenic risk scores for brain disorders (dementia, Parkinson’s disease, schizophrenia, and bipolar disorder) and diabetes (type 1 and type 2 diabetes). Model 4 was further adjusted for the Townsend Deprivation Index and educational attainment.

Rationing during the first 1000 days was associated with decelerated brain aging, as measured by BrainAGE. BrainAGE showed a modest positive correlation with chronological age (r = 0.488, *P* < 0.001), suggesting accelerated aging in older individuals (Fig. S11). Compared with the never-rationed reference group, sugar rationing exposure during the first 1000 days was associated with significantly decelerated BrainAGE (Table 2). In analyses with BrainAGE as the continuous outcome, the extended exposure corresponded to a reduction in brain-predicted age of approximately −0.39 years (fully adjusted model, β = −0.39, 95% CI: −0.52 to −0.26), an association that remained consistent across all covariate adjustment models. When brain aging was dichotomized (accelerated aging = 1), individuals exposed to rationing up to 1000 days had significantly lower odds of being classified as having accelerated brain aging (β = −0.18, 95% CI: −0.26 to −0.09), consistent with a protective effect. In contrast, exposure limited to the in utero period alone showed a consistent but non-significant trend toward slower brain aging in both the continuous (β = −0.18, 95% CI: −0.37 to 0.01) and discrete (β = −0.07, 95% CI: −0.19 to 0.05) analyses (Table 2). Sugar rationing during the first 1000 days was associated with a decelerated BrainAGE, with protective effects consistently observed across continuous and discrete brain aging metrics and all covariate adjustment models (Fig. 4). Sex-stratified analyses indicated broadly similar patterns across sexes, with somewhat stronger associations in females (Fig. S12). Exposure to rationing during the first 1000 days post-conception was associated with lower BrainAGE and reduced odds of accelerated brain aging in both females and males across all models. Inutero-only rationing showed consistent but non-significant associations with both outcomes in both sexes (Fig. S12).

**Table 2:** Association between early-life sugar rationing and brain aging

Type of brainaging measure	Exposure period	Model 1	Model 2	Model 3	Model 4
Continuous	Not rationing	Reference			
	Rationing in utero	−0.14 (−0.33, 0.04)	−0.18 (−0.37, 0.01)	−0.18 (−0.37, 0.01)	−0.18 (−0.37, 0.01)
	Rationing up to 1000 days	−0.36 (−0.49, −0.23)	−0.39 (−0.52, −0.26)	−0.39 (−0.52, −0.26)	−0.39 (−0.52, −0.26)
Discrete	Not Rationing	Reference			
	Rationing in utero	−0.06 (−0.17, 0.06)	−0.07 (−0.19, 0.05)	−0.07 (−0.19, 0.05)	−0.07 (−0.19, 0.05)
	Rationing up to 1000 days	−0.17 (−0.25, −0.08)	−0.17 (−0.26, −0.09)	−0.18 (−0.26, −0.09)	−0.18 (−0.26, −0.09)

Those who were never exposed to rationing (born after July 1954) served as the reference group. For continuous outcomes, linear regression coefficients (β) with 95% confidence intervals are reported. For binary outcomes (e.g., accelerated aging vs. non-accelerated), logistic regression coefficients (β) with 95% confidence intervals are reported. The crude model (Model 1) did not adjust for any covariates. Model 2 was adjusted for calendar month of birth, survey year, deciles of north and east birth coordinates, place of birth (England, Wales, or Scotland), self-reported sex, and race (White vs. non-White). Model 3 was additionally adjusted for genetic risks, including parental history of brain diseases (depression, dementia, or Parkinson’s disease) and polygenic risk scores for brain disorders (dementia, Parkinson’s disease, schizophrenia, and bipolar disorder) and diabetes (type 1 and type 2 diabetes). Model 4 was further adjusted for the Townsend Deprivation Index and educational attainment.

## Discussion

In this quasi-experimental study, we leveraged the natural experiment provided by the United Kingdom’s post-war sugar rationing policy to investigate the association between early-life sugar restriction and lifelong neuropsychiatric health. To our knowledge, this study provides quasi‑experimental evidence that restricted sugar availability during the first 1000 days of life is associated with reduced risks of both neurological and psychiatric disorders in adulthood. Compared with non-rationed individuals, those exposed to sugar restriction during the first 1000 days post-conception exhibited a 27% lower risk of dementia, a 46% reduction in AD risk, and decreased risks of depression (11%) and anxiety disorders (20%), with more pronounced benefits in females. Psychiatric benefits exhibited a dose-response pattern, becoming evident after 6 months of rationing exposure. Neuroimaging analyses further revealed that rationing during the first 1000 days was associated with larger subcortical volumes (e.g., putamen, hippocampus, thalamus), widespread reductions in gray–white matter intensity contrast, and a decelerated BrainAGE (approximately 0.39 years younger), indicating a younger-appearing brain. Collectively, these findings suggest a potentially critical window during which early-life sugar restriction may exert lasting protective effects on brain structure and the pace of neurobiological aging. This is consistent with the DOHaD hypothesis, which emphasizes the critical role of early-life environmental exposures (particularly during the first 1000 days) in shaping long-term health outcomes^18^.

Previous research has primarily focused on extreme metabolic disturbances such as gestational diabetes, linking maternal dysglycemia to impaired neurodevelopment in offspring^12,19^. However, our study extends this paradigm by examining population-level variations in sugar exposure using a quasi-experimental design. Notably, extended sugar restriction throughout the first 1000 days conferred stronger neuroprotective benefits than restriction limited to the prenatal period, underscoring the importance of sustained nutritional interventions across this developmental window. In line with prior evidence^15,20^, our results highlight the potential long‑term neurocognitive correlates of early‑life sugar exposure and highlight the need for further prospective studies in contemporary, diverse populations before any policy implications can be drawn. However, as with all quasi-experimental designs, these associations should not be interpreted as definitively causal. The natural experiment strengthened internal validity by creating a sharp policy-driven contrast, but unmeasured confounders tied to the post-war recovery period, secular trends in healthcare, and selective participation may still bias estimates.

The neuroprotective effects of early-life sugar restriction may operate through multiple biological pathways. First, in utero, the developing fetus is susceptible to maternal insulin and metabolic states, which can influence development through epigenetic, hormonal, and organ-reprogramming pathways^21^. Concurrently, reduced maternal intake of added sugars, including sucrose, may prevent fructose-induced metabolic dysregulation, thereby improving insulin sensitivity and reducing neuroinflammation^22^. Second, sugar restriction may also support normative epigenetic programming by avoiding hyperglycemia-induced disruptions to DNA methylation and histone modifications at genes critical for neurodevelopment, including those involved in synaptic plasticity and oxidative stress response^23^. Third, limiting sugar intake may attenuate maternal immune activation, reducing neurotoxic kynurenine production and oxidative stress, both of which can disrupt fetal neuronal migration^14,24^. Animal models further support these pathways: maternal high-sugar diets have been shown to impair N-methyl-D-aspartate receptor function and subunit composition, leading to deficits in prefrontal plasticity and cognitive function in offspring^25^. Together, these findings suggest that early-life sugar restriction preserves brain structure and function through coordinated effects on metabolic, inflammatory, epigenetic, and synaptic pathways^26^.

Structural preservation and decelerated neurobiological aging may represent potential neurobiological correlates linking early‑life sugar restriction to late‑life brain health, although the cross‑sectional nature of the imaging data precludes mechanistic inference. The observed volumetric preservation in key brain structures, including the putamen, hippocampus, and subcortical gray matter, likely contributes to the reduced risk of dementia observed in the cohort. The putamen, essential for motor coordination and involved in executive function, may serve as a neural substrate linking early nutritional environment to later-life cognitive resilience^27,28^. Hippocampal preservation supports memory processes, while maintained subcortical gray matter volume may enhance overall neural network integrity^29,30^. The directionality of gray–white matter contrast is context-dependent: it is elevated in obsessive-compulsive disorder, where it reflects cortical microstructural alterations, including abnormal intracortical myelination^31^, but reduced in our aging cohort. In our study, this reduction could reflect various microstructural alterations, such as changes in myelination, water content, or partial volume effects. While enhanced neural efficiency is one plausible interpretation, we cannot distinguish these possibilities with the current data, and this finding should therefore be considered hypothesis‑generating.

Consistent with these structural findings, the postnatal exposure group exhibited a decelerated BrainAGE of approximately −0.39 years, suggesting a younger-appearing brain on neuroimaging. This imaging metric is clinically meaningful, as prior work has shown that a larger BrainAGE (indicating an older-appearing brain) is associated with vascular burden, inflammation, and poorer cognitive performance^32^. Thus, the decelerated gap observed in our study is consistent with potentially delayed neurobiological aging, which in prior work has been associated with resilience against age-related cognitive decline and neurodegenerative diseases. However, direct evidence linking BrainAGE deceleration to improved cognitive outcomes in this cohort is limited^33^. Collectively, the multimodal MRI findings suggest that limiting added sugars in early development is associated with brain structural integrity and slows neurobiological aging. Of note, the healthy reference sample for brain age prediction was used exclusively to establish a normative aging trajectory, ensuring that the BrainAGE reflects deviations from physiological rather than pathological aging. This approach follows the established standard in the field^34^ and allows us to attribute observed BrainAGE differences to early-life sugar rationing rather than to pre-existing disease states.

Notably, the sex differences observed in our study, particularly the suggestion of stronger protective associations in females, merit further investigation into their underlying mechanisms. These differences may arise from interactions between sex hormones, metabolic pathways, and neurodevelopment^35^. Estrogen, for instance, exerts neuroprotective effects and modulates insulin sensitivity and inflammatory responses in the brain^36–38^. A low-sugar environment during early development might synergize with these endogenous protective mechanisms in females, amplifying long-term benefits. In males, benefits were largely confined to anxiety disorders, potentially reflecting increased susceptibility to oxidative stress and reduced neuroendocrine adaptive capacity^38,39^. These sex-specific patterns may also reflect differential vulnerability of developing neural circuits to nutritional insults, with males exhibiting heightened sensitivity to early-life metabolic perturbations.

While this study provides insights into the association between early-life sugar restriction and long-term brain health, we acknowledge several limitations. First, despite our quasi-experimental design and rigorous covariate adjustments, residual confounding cannot be fully excluded. The abrupt end of rationing coincided with broader post-war economic recovery, improved maternal healthcare, and changing infant feeding practices, which may have independently influenced neurodevelopment. Although sugar intake changed most dramatically, we cannot fully isolate its specific effect from these concurrent secular trends. Birth timing relative to the policy cutoff may correlate with seasonal nutritional variations (e.g., winter versus summer births) or maternal age patterns, which were only partially captured by our covariates. In our neuroimaging analyses, we did not adjust for hypertension, diabetes, smoking, or alcohol use in the primary models, as these factors may act as mediators rather than confounders in the pathway from early-life sugar exposure to brain structural outcomes; adjusting for them could introduce overadjustment bias. Nevertheless, we acknowledge that vascular and metabolic factors could partially mediate the observed associations. Second, while case ascertainment used multisource health records, the UK Biobank may underrepresent mild psychiatric disorders managed predominantly in community settings, possibly leading to under-ascertainment. Third, diagnostic accuracy varies across conditions; dementia is well-validated, whereas anxiety and depression rely more heavily on self-reports and primary care coding, which may be constrained by the cohort’s specific historical and demographic context. The UK Biobank participants, born around the 1950s in a post-war, predominantly White British setting, experienced unique nutritional and socioeconomic environments. The effects of early-life sugar restriction may differ in contemporary populations with ubiquitous high-sugar diets, different genetic backgrounds, or varied early-life care practices, necessitating replication in diverse modern cohorts. Fourth, we acknowledge that rationing ended for multiple food categories by July 1954; however, National Food Survey data showed that consumption of fats, cheese, meat, cereals, and overall protein exhibited only minor changes during our study window, whereas sugar intake increased sharply immediately after de-rationing^20,40,41^. This distinct pattern suggests that sugar was the primary dietary factor driving the observed associations. Nonetheless, we cannot completely exclude the possibility that concurrent, albeit small, changes in the intake of other nutrients may have contributed to the observed associations. Fifth, UK Biobank records parental history only for depression, dementia, and PD; history for other psychiatric conditions (e.g., anxiety disorders, bipolar disorder, and schizophrenia) is unavailable. While family history is the strongest predictor of psychiatric disorders in the next generation^42^, its absence for several conditions represents a limitation. To partially compensate, we included PRS for schizophrenia, bipolar disorder, and other conditions. As highlighted by Mollon et al., PRS for major psychiatric disorders show limited stability (inconsistent identification of high-risk individuals across different PRS versions) and uniformly low specificity (50.7–56.4%), capturing only a fraction of heritability^43^. However, the combined use of available parental history and PRS represents the most rigorous approach permitted by the data, and the stability of our estimates across models supports this approach. Finally, the cross-sectional nature of neuroimaging data and regional variations in detection sensitivity preclude definitive causal inferences and necessitate cautious interpretation of both positive and negative structural findings.

This historical natural experiment provides supportive evidence consistent with the hypothesis that early-life exposure to a lower-sugar environment is associated with long-term benefits for brain health, including lower risks of dementia and common mental disorders, potentially protective alterations in brain structure, and decelerated brain aging as quantified by BrainAGE. The study’s principal contribution lies in leveraging a policy shift to strengthen causal inference on developmental programming, while integrating neuroimaging to explore potential neurobiological substrates. These findings require replication in contemporary cohorts with detailed early-life dietary assessments before informing clinical or public health recommendations.

## Methods

### Study design and participants

This study leveraged data from the UK Biobank, a prospective cohort comprising more than 500,000 adults recruited between 2006 and 2010. The UK Biobank’s design and data collection have been reported previously^44^. To examine the long-term effects of limited sugar exposure during the first 1000 days post-conception on adult brain health, we adopted an event-study design that leveraged the abrupt termination of the UK’s sugar rationing policy in September 1953 as a natural experiment^45^. The analytic framework, including the delineation of the 1000-day window from conception and the application of regression discontinuity design, follows methods previously validated in studies using this quasi-experimental design^15,20^.

As illustrated in Fig. S1, which depicts sugar consumption trends from 1950 to 1956, sugar intake exhibited marked fluctuations during this post-war transition relative to rationing-era baselines. Leveraging this natural experiment, we included individuals conceived within a 1000-day window around this date (born October 1951 – March 1956), given that ages 0–3 years represent a critical period for brain development^46^. Since exact conception dates were unavailable, we estimated them from birth dates, assuming a uniform gestational period of 10 months. After excluding multiple births, adoptees, participants not born or residing in the UK, and those with missing data on geolocation, PRS, demographics, or parental history of brain disorders or depression, 60,394 participants remained for primary analyses of disease outcomes (sub-cohort 1). Additionally, among the 62,661 participants who underwent brain MRI, we identified a neuroimaging sub-cohort (sub-cohort 2) comprising 9,053 individuals who were born within the same window (October 1951 – March 1956) and had available T1-weighted MRI scans, no pre-existing brain disorders, and complete covariate data. This subsample, selected for its absence of pre‑existing brain disorders, was used to establish a normative brain aging trajectory. The disease and neuroimaging analyses address complementary questions: incident disease versus subclinical aging. The UK Biobank was reviewed and approved by the National Information Governance Board for Health and Social Care and the National Health Service Northwest Multicenter Research Ethics Committee (11/NW/0382). The study was conducted in accordance with the Declaration of Helsinki. All participants provided written informed consent.

### Assessment of early-life exposure to sugar rationing

Previous studies have shown that the end of UK sugar rationing in September 1953 led to a sustained doubling of daily adult sugar intake^15,17^. We classified participants by birth date relative to the rationing cessation. Participants born between October 1951 and June 1954, who were conceived within the 1000-day period preceding September 1953, were considered exposed to rationing. Whereas those born between July 1954 and March 1956 were classified as non-exposed. To capture critical developmental windows, we further divided exposure into three temporal categories: non-exposure (born July 1954 to March 1956), in-utero-only exposure (born October 1953 to June 1954), or exposure spanning both the prenatal and postnatal periods (born October 1951 to June 1954). Additionally, to concurrently assess potential dose-response relationships and sensitive periods in nutritional programming, postnatal exposure duration was subdivided into intervals of 6, 12, 18, and 24 months.

### Brain disorders outcomes

The primary outcomes comprised incident neurological and psychiatric disorders. Specifically, we examined dementia (including AD and vascular dementia), PD, depression, and anxiety disorders. Case ascertainment was based on the UK Biobank’s “First Occurrences” dataset (category 1712). Diagnostic information was extracted from the summary diagnoses of health outcomes (category 2002) and self-reported medical conditions (category 20002). Disease identification adhered to the International Classification of Diseases codes (ICD-9 and ICD-10). Dementia was defined as all-cause dementia (ICD-10: F00, F01, F02, F03, F05.1, F10.6, G30, G31 and I67.3), encompassing AD (F00 and G30), vascular dementia (F01 and I67.3), and other unspecified neurodegenerative dementias (F02–F03). PD was identified primarily via relevant primary and secondary diagnostic codes (G20–G21). Depression was defined using ICD-10 codes (F32–F33), and anxiety disorders using ICD-10 codes (F40–F41). Person-time of follow-up was calculated from the date of birth until the first recorded diagnosis, death, loss to follow-up, or the administrative censoring date (1 October 2022). Detailed phenotype definitions and corresponding ICD codes are provided in Table S1.

### Neuroimaging acquisition and processing

Secondary outcomes were derived from brain MRI. We used imaging-derived phenotypes (IDPs) directly provided by the UK Biobank. Specifically, the UK Biobank has implemented a standardized image processing pipeline on all brain imaging data, generating a comprehensive set of pre-computed IDPs available through the UK Biobank “Showcase” database. For our analyses, we used T1-weighted structural IDPs including global and regional gray matter volumes, white matter volumes, and subcortical structure volumes (e.g., hippocampus, thalamus), all processed with FSL tools (BET, FAST, SIENAX, FIRST) and FreeSurfer (version 6.0). From these T1-weighted structural sequences, the UK Biobank pipeline generated cortical surface parcellations (white and pial surfaces), subcortical segmentation, regional gray matter volumes, and gray–white matter intensity contrast. Subcortical structures were segmented using FreeSurfer’s automated pipeline, with further sub-segmentation (e.g., of hippocampal subfields). All these IDPs were generated through the UK Biobank’s centralized, quality-controlled pipeline, ensuring consistency across participants and eliminating the need for individual-level raw data processing. Detailed documentation of the acquisition protocols, processing pipelines, and IDP definitions is publicly available in the UK Biobank Brain Imaging Documentation^47,48^.

### Brain-predicted age gap estimation

To quantify individual differences in brain aging, we estimated the BrainAGE using a validated machine learning framework^49,50^. First, we established a healthy reference sample (*N* = 56,207) comprising individuals who had no recorded history of neurological or psychiatric disorders, stroke, traumatic brain injury, or brain tumor, and whose high-quality T1-weighted structural MRI data passing standardized quality control. This sample was randomly split into a training set (80%) and an internal test set (20%). We then employed least absolute shrinkage and selection operator (LASSO) regression with 10-fold cross-validation to model chronological age as a function of the image-derived phenotypes. The optimal regularization parameter (lambda) was selected as the value that minimized the cross-validated mean squared error. The final fitted model was applied to all participants in sub-cohort 2 to obtain their predicted brain age. The BrainAGE for each individual was then calculated as the predicted brain age minus the individual’s chronological age. A positive BrainAGE signifies an older-appearing brain (accelerated aging), while a negative value indicates a younger-appearing brain (delayed aging). In the statistical analyses, BrainAGE was treated in two complementary ways: (1) as a continuous outcome in linear regression models, and (2) as a binary outcome (accelerated aging: BrainAGE > 0 vs. BrainAGE ≤ 0) in logistic regression models.

### Assessment of covariates

We identified and adjusted for a range of potential individual-level confounders. In all analyses, we adjusted for a core set of baseline covariates, including sex, region of birth (England, Wales, Scotland), ethnicity (White/other), calendar month of birth, survey year, and deciles of the north and east birth coordinates. To further account for socioeconomic status, we additionally included the Townsend Deprivation Index (an area-based measure of material deprivation) and individual educational attainment (a well-established proxy for lifelong socioeconomic position). For familial and genetic confounding, we adjusted for parental history of depression, dementia, and PD, which are the only such variables systematically recorded in UK Biobank; history for other psychiatric conditions (e.g., anxiety, bipolar disorder, schizophrenia) is not available. In addition, we included polygenic risk scores (PRS) for dementia, PD, schizophrenia, bipolar disorder, and type 1 and type 2 diabetes. These PRS were derived from the UK Biobank’s official Polygenic Risk Score Release (Category 300). This release provides PRS for 28 diseases and 25 quantitative traits, computed via a unified pipeline by Genomics PLC (project 9659)^51^. Specifically, we used the Standard PRS set, which was trained on external GWAS summary data only and is available for all participants. PRS for anxiety and depression were not available. Further details on these measurements are available on the UK Biobank website (www.ukbiobank.ac.uk). A complete list of UK Biobank field IDs and detailed descriptions for all covariates is provided in Table S2.

### Statistical analysis

Baseline characteristics of the study cohorts were summarized using means (with standard deviations, SD) and medians (with interquartile ranges) for continuous variables, and frequencies (percentages) for categorical variables. Between-group comparisons were evaluated using Mann–Whitney U tests for continuous variables and chi-square (χ²) tests for categorical variables, as appropriate. Unadjusted differences in cumulative incidence were visualized with Kaplan–Meier curves and formally tested using the log-rank test. To estimate the association between early-life sugar rationing status and the risk of incident brain disorders, we fitted Cox proportional hazards models and reported HRs with 95% CIs. We constructed four nested models to sequentially account for potential confounding: Model 1 was unadjusted; Model 2 adjusted for core demographics (sex, birth region, ethnicity, birth month, and geographic coordinates); Model 3 further adjusted for parental history of depression, dementia, and PD, and relevant PRS (for dementia, PD, schizophrenia, bipolar disorder, and diabetes); and Model 4 additionally adjusted for the Townsend Deprivation Index and individual educational attainment. The proportional hazards assumption was verified by testing Schoenfeld residuals. To account for multiple comparisons across outcomes and imaging phenotypes, we controlled the false discovery rate (FDR) using the Benjamini–Hochberg procedure, with an FDR-adjusted *P* value < 0.05 deemed significant. For neuroimaging analyses, the fully adjusted model included age at MRI, sex, head motion, scanner position, calendar month of birth, deciles of north and east birth coordinates, the Townsend Deprivation Index, educational attainment, place of birth, ethnicity, and PRS for brain disorders. For volumetric traits, we additionally adjusted for total intracranial volume. We did not include hypertension, diabetes, smoking, or alcohol use as covariates in the primary neuroimaging models, as these factors are potential mediators rather than confounders in the causal pathway from early-life sugar exposure to brain aging; adjusting for them would introduce overadjustment bias. We did not adjust for depressive or anxiety disorders in the neuroimaging analyses, as both the imaging subsample and the brain age training sample were explicitly restricted to individuals without pre‑existing neurological or psychiatric conditions, rendering such adjustment unnecessary by design. All analyses were conducted using R statistical software (version 4.4.2). For primary analyses, a two-sided *P* value < 0.05 was considered statistically significant.

## Supplementary information

Supplementary Material

## Data Availability

Individual-level UK Biobank data (phenotypic, neuroimaging, and genotype) are available via the UKB Research Analysis Platform (RAP). Detailed information on registration and data access can be found at http://www.ukbiobank.ac.uk/register-apply/. For any further data requests, please contact the corresponding authors.
