# Supplementary material for: Early-life sugar rationing, brain aging, and long-term neurodegenerative and psychiatric health outcomes: a population-based natural experiment study
Source: NPJ Aging. 2026 Jul 22;12(1):155. doi: 10.1038/s41514-026-00452-z (PMC13754769; doi:10.1038/s41514-026-00452-z)
Supplement: Supplementary file 1 [file 41514_2026_452_MOESM1_ESM.doc]

**Early-life sugar rationing, brain aging, and long-term neurodegenerative and psychiatric health: A population-based natural experiment study**

**Supplemental M**aterials

| Table S1 | UK Biobank codes for brain disorders |  |  |
| --- | --- | --- | --- |
| Table S2 | The variables used in the present study from the UK Biobank |  |  |
| Table S3 | Association between rationing pattern and incident brain disorders in offspring in models with different sets of covariables and the fully adjusted model |  |  |
| Table S4 | The effect of rationing exposure on brain diseases stratified by gender |  |  |
| Table S5 | Baseline characteristics of participants for the neuroimaging subset in the UK Biobank |  |  |
| Figure S1 | The study design for the sugar rationing cohort in the UK Biobank |  |  |
| Figure S2 | The cumulative hazard (A) and risk table (B) of incident Alzheimer’s disease, stratified by exposure to rationing |  |  |
| Figure S3 | The cumulative hazard (A) and risk table (B) of incident dementia, stratified by exposure to rationing |  |  |
| Figure S4 | The cumulative hazard (A) and risk table (B) of incident Parkinson’s disease, stratified by exposure to rationing |  |  |
| Figure S5 | The cumulative hazard (A) and risk table (B) of incident anxiety disorders, stratified by exposure to rationing |  |  |
| Figure S6 | The cumulative hazard (A) and risk table (B) of incident depression disorders, stratified by exposure to rationing |  |  |
| Figure S7 | Association between rationing pattern and incident brain disorders relative to the hazard rate of never-rationed adults born July–December 1954 |  |  |
| Figure S8 | The effect of rationing pattern on traits from the cortical parcellation (destrieux atlas, a2009s) |  |  |
| Figure S9 | The effect of rationing pattern on white matter surface traits derived from the ex vivo Brodmann parcellation |  |  |
| Figure S10 | The effect of rationing pattern on white matter surface traits derived from the Desikan-Killiany-Tourville parcellation |  |  |
| Figure S11 | Comprehensive assessment of brain age prediction in healthy UK Biobank participants based on T1-weighted MRI data |  |  |
| Figure S12 | Sex stratified association between early-life sugar rationing and brain aging. |  |  |

**Table S1. UK Biobank codes for brain disorders**

| **Outcomes** | **Self-reported field code** | **International Classification of Diseases-10 (ICD-10)** | **International Classification of Diseases-9 (ICD-9)** |
| --- | --- | --- | --- |
| **Dementia** | 20002 (1263) | F00, F01, F02, F03, F05.1, F10.6, G30, G31, I67.3 | 290, 291.2, 294.1, 331 |
| **Alzheimer’s disease** | - | F00, G30, | 331.0 |
| **Parkinson’s disease** | 20002 (1262, 1848) | G20 | 332 |
| **Depressive disorder** | 20002 (1286, 1291, 1531) | F32, F33 | 311, 290.6, 296.2 |
| **Anxiety disorder** | 20002 (11287) | F40, F41 | 300.0–300.3 |

**Table S2. The variables used in the present study from the UK Biobank.**

| **Variables** | **Field ID** | **Measurements and Assessments** | **Value type** |
| --- | --- | --- | --- |
| **Age** | 21022 | A self-completed touch-screen questionnaire. | Continuous variable |
| **Sex** | 31 | A self-completed touch-screen questionnaire. | Categorical variable |
| **Ethnicity** | 21000 | A self-completed touch-screen questionnaire. | Categorical variable |
| **Calendar month of birth** | 52 | Verbal interview with a trained staff member at the assessment centre during the initial visit. | Continuous variable |
| **Country of birth** | 20115 | Verbal interview with a trained staff member at the assessment centre during the initial visit. | Categorical variable |
| **Place of birth (north co-ordinate)** | 129 | Latitude of birth location | Continuous variable |
| **Place of birth (east co-ordinate)** | 130 | Longitude of birth location | Continuous variable |
| **Education levels** | 6138 | Qualifications | Categorical |
| **Townsend deprivation index** | 22189 | Townsend deprivation index at recruitment | Continuous |
| **PRS for PD** | [26260](https://biobank.ndph.ox.ac.uk/showcase/field.cgi?tk=vC6Adb3YTzFpUZ1YtLZvA9wrHsRv8i7z497385&id=26260) | Polygenic score for the trait for all UK Biobank participants. Data supporting these scores were obtained either entirely from external GWAS data (the Standard PRS set) or from a combination of external and internal UK Biobank data | Continuous variable |
| [**PRS for AD**](https://biobank.ndph.ox.ac.uk/showcase/field.cgi?tk=vC6Adb3YTzFpUZ1YtLZvA9wrHsRv8i7z497385&id=26206) | [26206](https://biobank.ndph.ox.ac.uk/showcase/field.cgi?tk=vC6Adb3YTzFpUZ1YtLZvA9wrHsRv8i7z497385&id=26206) | Polygenic score for the trait for all UK Biobank participants. | Continuous variable |
| **PRS for bipolar disorder** | 26214 | Polygenic score for the trait for all UK Biobank participants | Continuous variable |
| [**PRS for type 1 diabetes**](https://biobank.ndph.ox.ac.uk/showcase/field.cgi?tk=vC6Adb3YTzFpUZ1YtLZvA9wrHsRv8i7z497385&id=26283) | [26283](https://biobank.ndph.ox.ac.uk/showcase/field.cgi?tk=vC6Adb3YTzFpUZ1YtLZvA9wrHsRv8i7z497385&id=26283) | Polygenic score for the trait for all UK Biobank participants. | Continuous variable |
| [**PRS for type 2 diabetes**](https://biobank.ndph.ox.ac.uk/showcase/field.cgi?tk=vC6Adb3YTzFpUZ1YtLZvA9wrHsRv8i7z497385&id=26283) | [26285](https://biobank.ndph.ox.ac.uk/showcase/field.cgi?tk=vC6Adb3YTzFpUZ1YtLZvA9wrHsRv8i7z497385&id=26283) | Polygenic score for the trait for all UK Biobank participants. | Continuous variable |
| **Standard PRS for schizophrenia** | [26275](https://biobank.ndph.ox.ac.uk/showcase/field.cgi?tk=vC6Adb3YTzFpUZ1YtLZvA9wrHsRv8i7z497385&id=26275) | Polygenic score for the trait for all UK Biobank participants. | Continuous variable |
| [**Illnesses of father**](https://biobank.ndph.ox.ac.uk/showcase/field.cgi?tk=vC6Adb3YTzFpUZ1YtLZvA9wrHsRv8i7z497385&id=20107) | [20107](https://biobank.ndph.ox.ac.uk/showcase/field.cgi?tk=vC6Adb3YTzFpUZ1YtLZvA9wrHsRv8i7z497385&id=20107) | A self-completed touch-screen questionnaire. | Categorical variable |
| [**Illnesses of mother**](https://biobank.ndph.ox.ac.uk/showcase/field.cgi?tk=vC6Adb3YTzFpUZ1YtLZvA9wrHsRv8i7z497385&id=20110) | [20110](https://biobank.ndph.ox.ac.uk/showcase/field.cgi?tk=vC6Adb3YTzFpUZ1YtLZvA9wrHsRv8i7z497385&id=20110) | A self-completed touch-screen questionnaire. | Categorical variable |

BMI, Body ass index; PD, Parkinson’s disease; AD, Alzheimer’s disease; PRS, Polygenic Risk Score.

**Table S3. Association between rationing pattern and incident brain disorders in offspring in models with different sets of covariables and the fully adjusted model**

| **Diseases** | **Rationing exposure** | **Event count** | **Hazard Ratio (95%CI)** | | | |
| --- | --- | --- | --- | --- | --- | --- |
|  |  |  | **Model 1** | **Model 2** | **Model 3** | **Model 4** |
| **Dementia** | No rationing | 102 | References | | | |
|  | Rationing in-utero | 43 | 0.78 (0.55, 1.11) | 0.78 (0.54, 1.12) | 0.77 (0.53, 1.11) | 0.76 (0.52, 1.10) |
|  | Rationing up to 1000 days | 205 | 0.76 (0.59, 0.98) | 0.75 (0.58, 0.97) | 0.73 (0.57, 0.95) | 0.73 (0.56, 0.94) |
| **AD** | No rationing | 44 | References | | | |
|  | Rationing in-utero | 15 | 0.56 (0.31, 1.01) | 0.56 (0.30, 1.04) | 0.55 (0.30, 1.02) | 0.55 (0.30, 1.02) |
|  | Rationing up to 1000 days | 79 | 0.61 (0.41, 0.91) | 0.58 (0.39, 0.88) | 0.56 (0.37, 0.84) | 0.55 (0.36, 0.82) |
| **PD** | No rationing | 88 | References | | | |
|  | Rationing in-utero | 61 | 1.31 (0.95, 1.82) | 1.38 (0.98, 1.94) | 1.38 (0.98, 1.94) | 1.40 (0.99, 1.97) |
|  | Rationing up to 1000 days | 202 | 1.00 (0.77, 1.29) | 1.01 (0.78, 1.31) | 1.01 (0.77, 1.31) | 1.01 (0.78, 1.32) |
| **Depressive disorder** | No rationing | 2603 | References | | | |
|  | Rationing in-utero | 1197 | 0.98 (0.92, 1.05) | 0.96 (0.90, 1.03) | 0.96 (0.89, 1.03) | 0.96 (0.89, 1.03) |
|  | Rationing up to 1000 days | 4412 | 0.91 (0.87, 0.95) | 0.90 (0.85, 0.94) | 0.90 (0.85, 0.94) | 0.89 (0.85, 0.94) |
| **Anxiety disorder** | No rationing | 1481 | References | | | |
|  | Rationing in-utero | 685 | 0.93 (0.85, 1.02) | 0.91 (0.83, 1.00) | 0.91 (0.83, 1.00) | 0.90 (0.82, 0.99) |
|  | Rationing up to 1000 days | 2530 | 0.82 (0.77, 0.88) | 0.81 (0.76, 0.86) | 0.81 (0.76, 0.86) | 0.80 (0.75, 0.85) |

AD, Alzheimer’s disease; PD, Parkinson’s disease. Note: The crude model (Model 1) did not adjust for any covariates. Model 2 was adjusted for calendar month of birth, survey year, deciles of north and east birth coordinates, place of birth (England, Wales, or Scotland), self-reported sex, and race (White vs. non-White). Model 3 was additionally adjusted for genetic risks, including parental history of brain diseases (depression, dementia, or Parkinson's disease) and polygenic risk scores for brain disorders (dementia, Parkinson's disease, schizophrenia, and bipolar disorder) and diabetes (type 1 and type 2 diabetes). Model 4 was further adjusted for the Townsend Deprivation Index and educational level.

**Table S4. The effect of rationing exposure on brain diseases stratified by gender**

| **Diseases** | **Gender** | **Rationing exposure** | **Hazard ratio (95%CI)** | | |  |
| --- | --- | --- | --- | --- | --- | --- |
|  |  |  | Model 1 | Model 2 | Model 3 | Model 4 |
| **Dementia** | Female | No rationing | 1.00 (References) | | | |
|  |  | Rationing in-utero | 0.74(0.44,1.27) | 0.73(0.42,1.26) | 0.72(0.41,1.24) | 0.68(0.39,1.18) |
|  |  | Rationing up to 1000 days | 0.80(0.56,1.16) | 0.78(0.54,1.13) | 0.76(0.53,1.10) | 0.74(0.51,1.07) |
|  | Male | No rationing | 1.00 (References) | | | |
|  |  | Rationing in-utero | 0.76(0.48,1.23) | 0.78(0.48,1.28) | 0.78(0.48,1.27) | 0.80(0.49,1.30) |
|  |  | Rationing up to 1000 days | 0.72(0.51,1.01) | 0.72(0.51,1.02) | 0.71(0.50,1.00) | 0.72(0.51,1.02) |
| **AD** | Female | No rationing | 1.00 (References) | | | |
|  |  | Rationing in-utero | 0.57(0.25,1.27) | 0.53(0.23,1.21) | 0.52(0.23,1.19) | 0.52(0.23,1.20) |
|  |  | Rationing up to 1000 days | 0.58(0.34,1.00) | 0.53(0.31,0.93) | 0.51(0.30,0.90) | **0.50(0.29,0.87)** |
|  | Male | No rationing | 1.00 (References) | | | |
|  |  | Rationing in-utero | 0.57(0.24,1.37) | 0.61(0.25,1.51) | 0.61(0.24,1.50) | 0.61(0.25,1.50) |
|  |  | Rationing up to 1000 days | 0.73(0.41,1.31) | 0.73(0.40,1.32) | 0.70(0.39,1.28) | 0.70(0.39,1.28) |
| **PD** | Female | No rationing | 1.00 (References) | | | |
|  |  | Rationing in-utero | 1.30(0.78,2.17) | 1.27(0.75,2.17) | 1.27(0.74,2.16) | 1.26(0.74,2.15) |
|  |  | Rationing up to 1000 days | 0.97(0.65,1.46) | 0.94(0.62,1.42) | 0.93(0.62,1.41) | 0.93(0.62,1.41) |
|  | Male | No rationing | 1.00 (References) | | | |
|  |  | Rationing in-utero | 1.21(0.79,1.84) | 1.31(0.85,2.03) | 1.31(0.85,2.03) | 1.34(0.87,2.09) |
|  |  | Rationing up to 1000 days | 0.96(0.69,1.33) | 0.99(0.71,1.37) | 0.99(0.71,1.37) | 1.00(0.72,1.40) |
| **Depressive disorder** | Female | No rationing | 1.00 (References) | | | |
|  |  | Rationing in-utero | 0.97(0.90,1.06) | 0.95(0.87,1.04) | 0.95(0.87,1.03) | 0.94(0.87,1.03) |
|  |  | Rationing up to 1000 days | 0.89(0.84,0.95) | 0.89(0.83,0.94) | 0.88(0.83,0.94) | **0.88(0.83,0.94)** |
|  | Male | No rationing | 1.00 (References) | | | |
|  |  | Rationing in-utero | 1.00(0.89,1.12) | 0.98(0.87,1.10) | 0.98(0.87,1.10) | 0.98(0.87,1.10) |
|  |  | Rationing up to 1000 days | 0.94(0.87,1.02) | 0.92(0.85,1.00) | 0.92(0.85,1.00) | 0.92(0.85,1.00) |
| **Anxiety disorder** | Female | No rationing | 1.00 (References) | | |  |
|  |  | Rationing in-utero | 0.99(0.88,1.10) | 0.96(0.86,1.08) | 0.96(0.86,1.07) | 0.95(0.85,1.07) |
|  |  | Rationing up to 1000 days | 0.84(0.78,0.91) | 0.83(0.77,0.90) | 0.83(0.77,0.90) | **0.83(0.76,0.90)** |
|  | Male | No rationing | 1.00 (References) | | |  |
|  |  | Rationing in-utero | 0.80(0.68,0.93) | 0.78(0.67,0.92) | 0.78(0.67,0.92) | **0.77(0.65,0.91)** |
|  |  | Rationing up to 1000 days | 0.77(0.69,0.86) | 0.76(0.68,0.85) | 0.76(0.68,0.85) | **0.75(0.67,0.83)** |

AD, Alzheimer’s disease; PD, Parkinson’s disease. Note: The crude model (Model 1) did not adjust for any covariates. Model 2 was adjusted for calendar month of birth, survey year, deciles of north and east birth coordinates, place of birth (England, Wales, or Scotland), self-reported sex, and race (White vs. non-White). Model 3 was additionally adjusted for genetic risks, including parental history of brain diseases (depression, dementia, or Parkinson's disease) and polygenic risk scores for brain disorders (dementia, Parkinson's disease, schizophrenia, and bipolar disorder) and diabetes (type 1 and type 2 diabetes). Model 4 was further adjusted for the Townsend Deprivation Index and educational level.

**Table S5. Baseline characteristics of participants for the neuroimaging subset in the UK Biobank**

| **Characteristics** | **Not**  **Rationing** | **Rationing**  **in-utero** | **Rationing up to 1000 days** | **P values** |
| --- | --- | --- | --- | --- |
| **N** | 3399 | 1515 | 5654 |  |
| **Sex, Male (%)** | 1505 (44.3) | 690 (45.5) | 2574 (45.5) | 0.482 |
| **Age (years), mean (sd)** | 64.12 (2.62) | 65.28 (2.63) | 66.27 (2.70) | <0.001 |
| **Ethnic, White (%)** | 3386 (99.6) | 1507 (99.5) | 5625 (99.5) | 0.644 |
| **Birth place (%)** |  |  |  | 0.452 |
| England | 3009 (88.5) | 1339 (88.4) | 5047 (89.3) | |
| Wales | 96 (2.8) | 34 (2.2) | 138 (2.4) |  |
| Scotland | 294 (8.6) | 142 (9.4) | 469 (8.3) |  |
| **Educational attainment, n (%)** |  |  |  | 0.518 |
| College education | 1608 (47.3) | 702 (46.3) | 2619 (46.3) |  |
| Vocational qualification | 135 (4.0) | 71 (4.7) | 275 (4.9) |  |
| Any school degree | 1464 (43.1) | 663 (43.8) | 2431 (43.0) |  |
| Other | 192 (5.6) | 79 (5.2) | 329 (5.8) |  |
| **TDI, mean (SD)** | -1.97 (2.66) | -2.09 (2.61) | -2.00 (2.63) | 0.377 |
| **Parents with PD (%)** | 324 (9.5) | 144 (9.5) | 559 (9.9) | 0.82 |
| **Parents with depression (%)** | 370 (10.9) | 163 (10.8) | 594 (10.5) | 0.845 |
| **Parents with stroke (%)** | 819 (24.1) | 380 (25.1) | 1526 (27.0) | 0.008 |
| **Parents with dementia (%)** | 437 (12.9) | 208 (13.7) | 797 (14.1) | 0.249 |
| **PRS for AD, mean (SD)** | 0.05 (0.99) | -0.01 (0.95) | 0.04 (0.99) | 0.126 |
| **PRS for PD, mean (SD)** | -0.13 (1.05) | -0.19 (0.99) | -0.14 (1.01) | 0.125 |
| **PRS for schizophrenia, mean (SD)** | -0.35 (1.02) | -0.35 (0.97) | -0.35 (0.97) | 0.998 |
| **PRS for bipolar disorder, mean (SD)** | -0.27 (1.02) | -0.22 (1.04) | -0.25 (1.02) | 0.274 |
| **PRS for type 1 diabetes, mean (SD)** | 0.07 (1.11) | 0.05 (1.07) | 0.06 (1.10) | 0.828 |
| **PRS for type 2 diabetes, mean (SD)** | -0.19 (0.97) | -0.16 (0.98) | -0.18 (0.96) | 0.581 |

TDI, Townsend Deprivation Index; SD, Standard Deviation; PD, Parkinson’s disease; AD, Alzheimer’s disease; PRS, Polygenic Risk Score.

# **Figure S1. The study design for the sugar rationing cohort in the UK Biobank.** The United Kingdom lifted its sugar rationing policy in September 1953, marking a significant step in postwar economic recovery. Adults conceived in the 1000 days prior to September 1953 were defined as “rationing up to 1000 days” (born October 1951 to June 1954) and adults conceived after as “non-rationed” (born July 1954 to March 1956). Adults exposed to rationing in utero were those born October 1953 to June 1954. In this study, the sugar consumption metrics are publicly available data sourced from the National Diet and Nutrition Survey (https://www.gov.uk/government/collections/national-diet-and-nutrition-survey).

**Figure S2.** **The cumulative hazard (A) and risk table (B) of incident Alzheimer’s disease, stratified by exposure to rationing.** (A) depicts crude cumulative risk and 95% confidence intervals, stratified by rationing pattern (no rationing, red; rationing in utero, blue; rationing up to 1000 days, green). *P* values were calculated using the log-rank test to examine differences between survival curves. (B) shows the cumulative number of events per rationing group.

**Figure S3.** **The cumulative hazard (A) and risk table (B) of incident dementia, stratified by exposure to rationing.** (A) depicts crude cumulative risk and 95% confidence intervals, stratified by rationing pattern (no rationing, red; rationing in utero, blue; rationing up to 1000 days, green). *P* values were calculated using the log-rank test to examine differences between survival curves. (B) shows the cumulative number of events per rationing group.

**Figure S4.** **The cumulative hazard (A) and risk table (B) of incident Parkinson’s disease, stratified by exposure to rationing.** (A) depicts crude cumulative risk and 95% confidence intervals, stratified by rationing pattern (no rationing, red; rationing in utero, blue; rationing up to 1000 days, green). *P* values were calculated using the log-rank test to examine differences between survival curves. (B) shows the cumulative number of events per rationing group.

**Figure S5.** **The cumulative hazard (A) and risk table (B) of incident anxiety disorders, stratified by exposure to rationing.** (A) depicts crude cumulative risk and 95% confidence intervals, stratified by rationing pattern (no rationing, red; rationing in utero, blue; rationing up to 1000 days, green). *P* values were calculated using the log-rank test to examine differences between survival curves. (B) shows the cumulative number of events per rationing group.

**Figure S6.** **The cumulative hazard (A) and risk table (B) of incident depression disorders, stratified by exposure to rationing.** (A) depicts crude cumulative risk and 95% confidence intervals, stratified by rationing pattern (no rationing, red; rationing in utero, blue; rationing up to 1000 days, green). *P* values were calculated using the log-rank test to examine differences between survival curves. (B) shows the cumulative number of events per rationing group.

**Figure S7.  Association between rationing pattern and incident brain disorders relative to the hazard rate of never-rationed adults born July–December 1954.** Each point represents a hazard ratio comparing adults exposed to rationing in utero or in utero plus 6/12/18/24 months after birth to the reference group (adults born July–December 1954). The model was adjusted for calendar month of birth, survey year, deciles of north and east birth coordinates, place of birth (England, Wales, or Scotland), self-reported sex, race (White vs. non-White), Townsend Deprivation Index, educational attainment, genetic risks (parental history of depression, dementia, stroke, or Parkinson’s disease), and polygenic risk scores for brain disorders (dementia, Parkinson’s disease, schizophrenia and bipolar disorder) and diabetes (type 1 and type 2 diabetes).

**Figure S8. The effect of rationing pattern on traits from the cortical parcellation (destrieux atlas, a2009s**)**.** The model was adjusted for head size, scanner site coordinates, calendar month of birth, deciles of north and east birth coordinates, place of birth (England, Wales, or Scotland), race (White vs. non-White), Townsend Deprivation Index, educational attainment, and genetic risks, including parental history of brain diseases (dementia, stroke, or Parkinson’s disease) and polygenic risk scores for brain disorders (dementia, Parkinson’s disease, schizophrenia, bipolar disorder, and stroke). Additionally, we adjusted for the presence of neurological disorders, psychiatric and behavioral disorders, and stroke at the time of MRI scan. Statistical significance was determined using the Benjamini-Hochberg method for false discovery rate correction.

**Figure S9. The effect of rationing pattern on white matter surface traits derived from the ex vivo Brodmann parcellation.** The model was adjusted for head size, scanner site coordinates, calendar month of birth, deciles of north and east birth coordinates, place of birth (England, Wales, or Scotland), race (White vs. non-White), Townsend Deprivation Index, educational attainment, and genetic risks, including parental history of brain diseases (dementia, stroke, or Parkinson’s disease) and polygenic risk scores for brain disorders (dementia, Parkinson’s disease, schizophrenia, bipolar disorder, and stroke). Additionally, we adjusted for the presence of neurological disorders, psychiatric and behavioral disorders, and stroke at the time of MRI scan. Statistical significance was determined using the Benjamini-Hochberg method for false discovery rate correction.

**Figure S10. The effect of rationing pattern on white matter surface traits derived from the Desikan-Killiany-Tourville parcellation.** The model was adjusted for head size, scanner site coordinates, calendar month of birth, deciles of north and east birth coordinates, place of birth (England, Wales, or Scotland), race (White vs. non-White), Townsend Deprivation Index, educational attainment, and genetic risks, including parental history of brain diseases (dementia, stroke, or Parkinson’s disease) and polygenic risk scores for brain disorders (dementia, Parkinson’s disease, schizophrenia, bipolar disorder, and stroke). Additionally, we adjusted for the presence of neurological disorders, psychiatric and behavioral disorders, and stroke at the time of MRI scan. Statistical significance was determined using the Benjamini-Hochberg method for false discovery rate correction.

**Figure S11. Comprehensive assessment of brain age prediction in healthy UK Biobank participants** **based on T1-weighted MRI data.** **(A)** Age distribution of the healthy sample (N = 56,273). **(B)** Scatter plot of actual versus brain-predicted age in the test set, with linear fit (red line) and identity line (dashed grey). Model performance: R^2^ = 0.774, MAE = 2.89 years. **(C)** Histogram of brain age gap (predicted age minus actual age). Vertical dashed lines indicate zero (grey), +1 year (red), and –1 year (blue) thresholds; proportions of accelerated (>0 year) and delayed (< 0 year) aging are annotated. **(D)** Brain age gap as a function of chronological age, with linear regression line (red) and 95% confidence band. Pearson correlation r = 0.488 (p < 0.001). Overall, the LASSO regression model applied to T1-weighted imaging features demonstrated robust prediction accuracy and revealed subtle age-related brain changes in the healthy population.

**Figure S12. Sex stratified** **association between early-life sugar rationing and brain aging.** For continuous outcomes, linear regression coefficients (β) with 95% confidence intervals are reported. For binary outcomes (e.g., accelerated aging vs. non-accelerated), logistic regression coefficients (β) with 95% confidence intervals are reported. The crude model (Model 1) did not adjust for any covariables. Model 2 was adjusted for calendar month of birth, survey year, deciles of north and east birth coordinates, place of birth (England, Wales, or Scotland), and race (White vs. non-White). Model 3 was additionally adjusted for genetic risks, including parental history of brain diseases (dementia, or Parkinson’s disease) and polygenic risk scores for brain disorders (dementia, Parkinson’s disease, schizophrenia, and bipolar disorder) and diabetes (type 1 and type 2 diabetes). Model 4 was further adjusted for the Townsend Deprivation Index and educational attainment.
